# From Polydeoxyribonucleotides (PDRNs) to Polynucleotides (PNs): Bridging the Gap Between Scientific Definitions, Molecular Insights, and Clinical Applications of Multifunctional Biomolecules

**DOI:** 10.3390/biom15010148

**Published:** 2025-01-19

**Authors:** Cíntia Marques, Alexandre Porcello, Marco Cerrano, Farid Hadjab, Michèle Chemali, Kelly Lourenço, Basste Hadjab, Wassim Raffoul, Lee Ann Applegate, Alexis E. Laurent

**Affiliations:** 1 Development Department, LOUNA REGENERATIVE SA, CH-1207 Geneva, Switzerland; c.marques@louna-aesthetics.com (C.M.); a.porcello@louna-aesthetics.com (A.P.); k.lourenco@louna-aesthetics.com (K.L.); 2 Aesthetic Surgery Department, Clinique Entourage, CH-1003 Lausanne, Switzerland; m.cerrano@entourage.ch; 3 Development Department, Albomed GmbH, D-90592 Schwarzenbruck, Germany; f.hadjab@albomed.eu; 4 Plastic and Aesthetic Surgery Service, Centre Médical Lausanne Ouest, CH-1008 Prilly, Switzerland; m.chemali@cmlo.ch; 5 Independent Consultant Office, F-74330 Poisy, France; abdelbasste@yahoo.fr; 6 Plastic and Reconstructive Surgery Service, Ensemble Hospitalier de la Côte, CH-1110 Morges, Switzerland; wassim.raffoul@ehc.vd.ch; 7 Regenerative Therapy Unit, Lausanne University Hospital, University of Lausanne, CH-1066 Epalinges, Switzerland; lee.laurent-applegate@chuv.ch; 8 Center for Applied Biotechnology and Molecular Medicine, University of Zurich, CH-8057 Zurich, Switzerland; 9 Oxford OSCAR Suzhou Center, Oxford University, Suzhou 215123, China; 10 Manufacturing Department, LAM Biotechnologies SA, CH-1066 Epalinges, Switzerland; 11 Manufacturing Department, TEC-PHARMA SA, CH-1038 Bercher, Switzerland

**Keywords:** anti-aging, DNA fragments, molecular weight, nomenclature, physicochemical properties, polydeoxyribonucleotides (PDRNs), polynucleotides (PNs), skin regeneration, standardization, wound healing

## Abstract

Polydeoxyribonucleotides (PDRNs) and polynucleotides (PNs) are similar DNA-derived biopolymers that have garnered significant scientific attention since the 1990s for their potential applications in wound healing and skin rejuvenation. These biopolymers exhibit a broad molecular weight (MW) range, typically spanning from 50 to 1500 kDa. However, recent studies have expanded this range to encompass fragments as small as 1 kDa and as large as 10,000 kDa. Clinically, PDRN/PN formulations, commercially available in various galenic forms (gels, creams, serums, masks, and injectables), have demonstrated promising effects in significantly promoting skin regeneration, reducing inflammation, improving skin texture, preventing scar formation, and mitigating wrinkles. Importantly, despite their widespread use in cosmetology and aesthetic dermatology, the interchangeable use of the terms “PDRN” and “PN” in the scientific literature (to describe polymers of varying lengths) has led to considerable confusion within the medical and scientific communities. To specifically address this PDRN/PN ambiguity, this narrative review proposes a standardized structure-based nomenclature for these DNA-derived polymers, the “Marques Polynucleotide Cutoff”, set at 1500 kDa. Thus, we propose that the term “PDRN” should be exclusively reserved for small- and medium-chain polymers (MW < 1500 kDa), while the term “PN” should specifically be used to denote longer-chain polymers (MW ≥ 1500 kDa). In a broader perspective, this classification is based on the distinct physicochemical properties and therapeutic effects of these DNA fragments of various MWs, which are comprehensively discussed in the present review.

## 1. Introduction

Polydeoxyribonucleotides (PDRNs) and polynucleotides (PNs) are terms that are used to designate a polymer composed of several units of deoxyribonucleotides. These biopolymers can notably be applied to promote wound healing and foster skin anti-aging effects [1,2,3,4,5,6]. In the past, these DNA fragments were described as having structures between 50 and 2000 base pairs (bp) [7,8]. Generally, the recent scientific literature considers that these polymers present a molecular weight (MW) ranging from 50 to 1500 kDa [2,4,9]. Notwithstanding, recent works describe such DNA fragments, used for aesthetic purposes, as short as 1 kDa [10] and as long as 10,000 kDa [11]. Of note, there are several PDRN/PN-based commercial products on the global market, such as gels, creams, serums, masks, and injectables [2]. These formulations are commonly used to promote skin regeneration and cutaneous anti-aging, by reducing hyperpigmentation, reducing the local oxidative/inflammatory state, improving skin texture, preventing scar formation, promoting hair regeneration, and exerting anti-wrinkle effects [2,3]. Overall, within the aesthetic and cosmeceutical domains, the terms “PDRN” and “PN” have been interchangeably used to refer to various DNA fragments [2,3,8,12,13], which has caused specific nomenclature confusion in the medical and scientific communities.

Based on this specific observation, the present narrative review highlights the importance of standardizing the nomenclature for these DNA fragments of distinct MW, as their respective physicochemical properties can lead to differences in cutaneous therapeutic effects. This review thus proposes, for the first time, a standardized structure-based nomenclature description for the “PDRN” and “PN” terms. To this end, we introduce the “Marques Polynucleotide Cutoff” value, which was set at a MW of 1500 kDa based on structural, functional, and historical elements. Simply put, for aesthetic and cosmeceutical purposes, the “PDRN” term should strictly be used to specifically describe small and medium chains of deoxyribonucleotides (i.e., MW < 1500 kDa, under the cutoff value), while the “PN” term should only be used to refer to longer chains of deoxyribonucleotides (i.e., MW ≥ 1500 kDa, over the cutoff value). For the sake of clarity, these definitions were applied in the various considerations and data reviews presented herein, in particular when discussing PDRN/PN therapeutic actions in cutaneous applications. From a structural viewpoint, the present narrative review will address the scientific definitions, sourcing and extraction modalities, molecular insights, and cutaneous clinical applications of these multifunctional biomolecules.

## 2. Brief Historical Context for PDRN/PN Biopolymers

**Figure 1:**
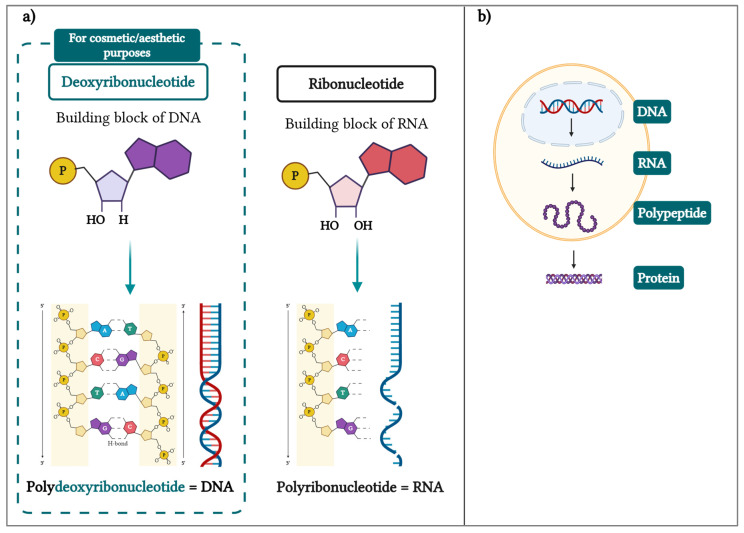
(**a**) Structure of DNA and RNA molecules, highlighting the chemical group differences on the deoxyribose sugar (DNA) and the ribose sugar (RNA). (**b**) Flow sequence of genetic information from DNA to proteins. Firstly, DNA serves as a template to produce RNA (i.e., transcription), which is then translated into polypeptides that fold into functional proteins. DNA—deoxyribonucleic acid; RNA—ribonucleic acid.

It is well known that polydeoxyribonucleotides (PDRNs) are composed (i.e., as the name indicates) of several deoxyribonucleotide units. These, in turn, constitute the building blocks of a larger double helix polymer, namely deoxyribonucleic acid (DNA), the basis of bioinformation storage and transmission [14]. Importantly, DNA is part of a bigger family (i.e., the polynucleotides), which also includes polyribonucleotides or ribonucleic acid (RNA; Figure 1) [15].

Specifically, DNA is located in the cell nucleus and includes all the genetic information of the biological organism, which is transformed by transcription into RNA (Figure 1b). Thereafter, RNA works as an intermediate, traveling into the cytosol, where the information can be translated into proteins (i.e., translation phase; Figure 1b) [16].

Due to their utmost functional importance in cells, both DNA and RNA have been the object of great interest from the scientific community and the object of numerous scientific publications over the last decades. In the 1990s, several studies notably reported the potential application of PDRN for wound healing enhancement, since the available evidence showed its efficacy in promoting fibroblast proliferation [8,17,18]. Such attributes were correlated with the PDRN-mediated activation of A_2A_ receptors and the consequent stimulation of the wound healing process (see Section 3) [7,19]. At the same time, other authors were able to establish a reliable extraction process for PDRN from human placenta [20], which eventually culminated with the approval of the first drug including PDRN as an active principle in 1994 (i.e., Placentex^®^; Mastelli, Sanremo, Italy) [21]. This product was initially approved as an injectable solution for dystrophic or dystrophic–ulcerative connective tissue disorders (i.e., against scarring, as an antidystrophic agent). For current manufacturing applications, however, PDRN is primarily sourced from salmon sperm, mainly harvested from chum salmon (*Oncorhynchus keta*) and rainbow trout (*Oncorhynchus mykiss*) [2].

Due to its aforementioned intrinsic wound healing properties, PDRN is also used for medicalized cosmetic and aesthetic purposes, mainly to reverse the signs of the skin aging process [2,3,8,12,13]. Of note, in the publications discussing the skin regeneration properties of this polymer, it is possible to find the terms “PDRN” and “PN” being used as synonyms [8]. In more recent works, the term “PDRN” is usually applied to describe smaller polymer chains and “PN” to describe longer polymers [22,23,24,25]. Importantly, these shifts in polymer names have accompanied the evolution of the extraction process for the DNA fragments of interest, as modern methods enable us to obtain longer chains and to better control the fragment MW distributions (see Section 4). In parallel, these evolutions were accompanied by new formulations, namely PN-based hydrogels, which have been widely used clinically, and will be further discussed in Section 6.

It is of the highest importance to note that, for aesthetic or cosmetic purposes, both the names “PDRN” and “PN” always refer to a chain of deoxyribonucleotides, meaning that the polymer is always of DNA origin. As stated before and for the sake of clarity, the term “PDRN” is going to be used in this review to describe small and medium chains of deoxyribonucleotides (i.e., MW < 1500 kDa) and the term “PN” is going to be used to refer to long chains of deoxyribonucleotides (i.e., MW ≥ 1500 kDa; see Section 5), in accordance with the defined “Marques Polynucleotide Cutoff” value of 1500 kDa.

## 3. PDRN/PN and Skin Regeneration

**Figure 2:**
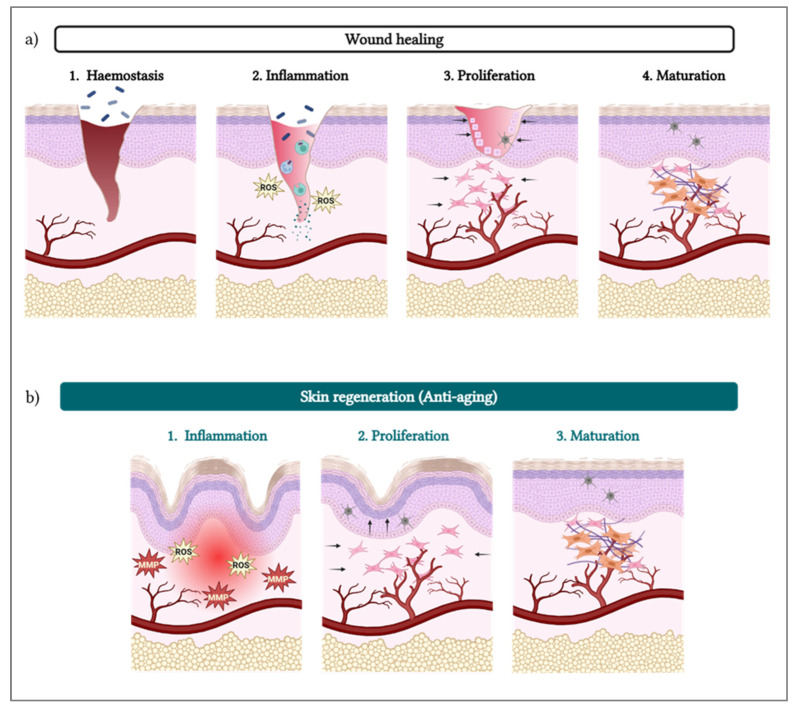
Similarities and differences between cutaneous wound healing and skin anti-aging mediated by PDRN/PN action. (**a**) The wound healing process is constituted by four main steps (hemostasis, inflammation, proliferation, and maturation). (**b**) In the case of anti-aging treatments, three steps of this process are transposable with the wound healing process (inflammation, proliferation, and maturation steps). MMP—matrix metalloproteinase; PDRN—polydeoxyribonucleotide; PN—polynucleotide; ROS—reactive oxygen species.

Due to their potent wound healing stimulation properties [26,27,28,29,30,31,32,33], PDRNs/PNs have been extensively applied for skin regeneration as cosmeceutical ingredients [5] and for medicalized aesthetic purposes. Of note, cutaneous aging processes involve similar dynamics to those of skin wounds and the related repair processes [34]. Therefore, the cellular pathways stimulated by PDRN/PN in wound healing also play a role in reversing the skin aging process, with the potential to regenerate cutaneous tissue that has already been lost (Figure 2).

**Figure 3:**
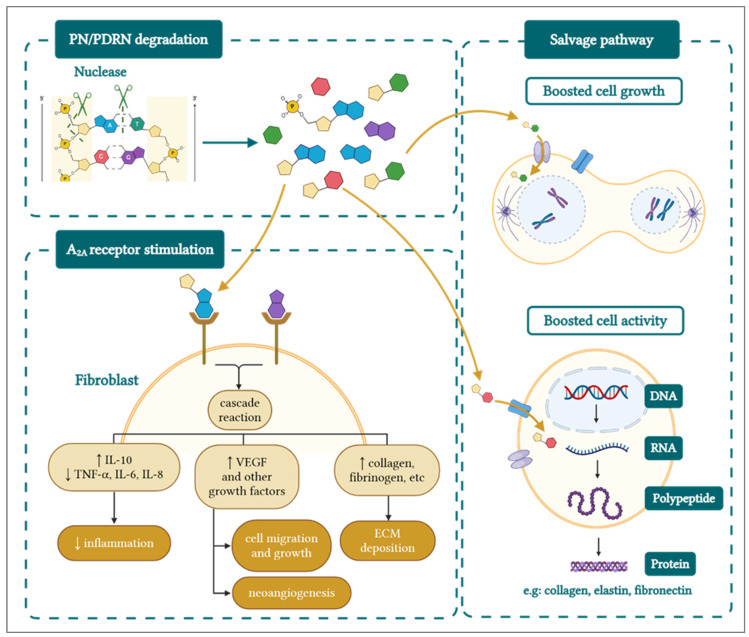
In order to be converted to active forms, PN and PDRN are degraded by endogenous nucleases, producing nucleotides. The nucleotides then bind to the A_2A_ receptors in fibroblasts, stimulating a cascade reaction that decreases inflammation, stimulates cell migration and growth, angiogenesis, and the maturation of the ECM. In parallel, the nucleotides can be recycled through the salvage pathway, accelerating cell growth and protein production by the cells, which can include collagen, elastin, and fibronectin. ECM—extracellular matrix; IL—interleukin; PDRN—polydeoxyribonucleotide; PN—polynucleotide; TNF—tumor necrosis factor; VEGF—vascular endothelial growth factor.

Nowadays, it is known that PDRNs/PNs stimulate skin regeneration through two main mechanisms: (i) stimulation of A_2A_ receptors in fibroblasts and (ii) material supply to the salvage pathway, which has been thoroughly reviewed elsewhere [3,5,6]. In this context, it is highly important to note that the first necessary step for PDRN/PN activity is their degradation or enzymatic breakdown. Therefore, by presenting a DNA-like structure, PDRNs/PNs are naturally degraded in situ by endogenous nucleases [9]. The generated units (i.e., deoxyribonucleotides) may then bind to the A_2A_ receptors in fibroblasts [7], which in turn stimulate several cellular pathways, as shown in Figure 3.

The three main activities mediated by PDRN/PN via A_2A_ receptor binding, as illustrated in Figure 3, may be summarized as follows:

• Inflammation resolution: The resulting cascade reaction leads to a decrease in the levels of pro-inflammatory cytokines (e.g., TNF-α, IL-6, IL-8) [7] and an increase in their anti-inflammatory counterparts (e.g., IL-10), decreasing the overall inflammatory status. The cascade also notably inhibits the synthesis and secretion of collagenase by synovial fibroblasts [7].
• Proliferation: Nucleotides stimulate the secretion of VEGF, which stimulates the formation of new blood vessels (i.e., neo-angiogenesis), as well as growth factors that stimulate cell migration and growth (i.e., fibroblasts and endothelial cells) [6,7,19,33,35,36].
• Remodeling: With decreased inflammation, increased blood support, and cell growth stimulation, the cells (e.g., fibroblasts) are surrounded by optimal conditions to produce collagen (i.e., types I and III) [37,38,39], elastin, and fibrinogen [18,33]. Those proteins then contribute to form the ECM, providing mechanical and structural support to fibroblasts, generating new tissue [27].

Importantly, the salvage pathway works in synergy with fibroblast activation through PDRN/PN binding to the A_2A_ receptors (Figure 3), positively controlling the three key PDRN/PN activities described hereabove [3,5,6]. Specifically, after PDRN/PN degradation into deoxyribonucleotides, the resulting units can be directly uptaken by the cells for anabolic purposes. Therein, the nucleotides are recycled in the cell through a series of pathways [6,9], which ultimately speed up the synthesis of ECM proteins by fibroblasts, by decreasing the energy necessary to produce those proteins (i.e., including collagen, fibrinogen, and elastin; Figure 3).

From a clinical standpoint, dermis regeneration is one of the most important targets to effectively manage skin aging signs [40,41], since it is well known that cutaneous collagen levels decrease with age [42]. However, the skin is also constituted by the subcutaneous layer and the epidermis [43], which are also important to consider to improve overall skin quality. Of note, there is evidence of PDRN efficacy on the stimulation of pre-adipocyte growth [44], which might suggest that it can reverse the loss of fat pad volume within the aging process [45]. Finally, PDRN can also be applied into the epidermis to control facial erythema [35] and to reduce hyperpigmentation. Therein, an in vitro study showed that PDRN decreases melanin biosynthesis in cultured skin cells [46], while its efficacy to reduce skin erythema is probably due to the polymer’s anti-inflammatory actions [47,48]. As a side note, there is even evidence that PDRN can induce an increase in osteoblast growth [49]. Thus, PDRN stimulates skin regeneration and improves its quality by stimulating multiple cutaneous layers, which underscores the polyvalent functional attributes of this biomolecule.

Moreover, the greatest functional advantages of PDRN lie in its intrinsic potential for inflammation resolution and full bio-resorption. The classic biostimulators on the commercial market, namely calcium hydroxylapatite (CaHA), poly-L-lactic acid (PLLA), and polycaprolactone (PCL), rely on an induced local inflammatory response to stimulate fibroblasts into producing collagen [50,51,52,53,54]. While the clinical knowledge about these products has extensively increased (i.e., greatly improving their safety), an incorrect administration of these biostimulators (e.g., CaHA, PLLA, PCL) might adversely lead to granuloma formation [51,55,56,57]. Furthermore, while granulomas have been reported for all kinds of fillers, hyaluronic acid (HA)-based interventions can be easily reversed by hyaluronidases, while CaHA, PLLA, and PCL fillers might eventually require surgical excision [56,57,58]. Thus, PDRN is fundamentally different from traditional biostimulators, since it induces protein production by decreasing inflammation, with virtually no risk of nodule formation.

To the best of our current knowledge, there are no reported cases of nodule or granuloma formation after PN-based filler injections. Interestingly, a clinical case of long-term complications for a polymethyl–methacrylate filler (i.e., for over 20 years) was reported, including acute edema progressing to skin dystrophia and persistent cutis laxa [59]. In the same patient, an HA-PN filler was effectively used to address these skin laxity issues (i.e., resulting from the polymethyl–methacrylate filler sequelae). Overall, it may thus be set forth herein, based on the available scientific and clinical evidence, that PDRNs/PNs not only serve as biostimulators, but mediate global tissular regeneration, including the formation of blood vessels, cell growth, and protein production.

## 4. PDRN/PN Sourcing and Extraction Methodologies

Multiple sources and extraction processes have been reported for PDRN/PN obtention in a sterile and injectable-grade form. According to the summary of the product characteristics of Placentex^®^, PDRN is thermoresistant, meaning that it is possible to sterilize it through classical moist heat treatment procedures in an autoclave at 121 °C, ensuring maximum microbiological safety [21]. In particular, high-temperature sterilization ensures a very high percentage of DNA (i.e., up to 90% purity) and the presence of low amounts of free amino acids, low-MW peptides, and glycosaminoglycans [7,19]. While this process is efficient for obtaining pure PDRN polymers with chain lengths ranging between 50 and 2000 bp [7], it is difficult to control the final size of the obtained polymer. Notwithstanding, as early as the 1990s, a patent detailed a more complex extraction process which would allow the upscaling of pharmaceutical-grade PDRN production [20].

In addition to the initial treatment with high temperatures, the extraction process includes a proteolysis step, three separation steps, and controlled partial depurination of the native PDRN. Since PDRN is made up of DNA, it fundamentally bears an informational function; thus, the depurination steps add an extra layer of safety by eliminating the polymer’s informational capability (i.e., limiting the transfer of genetic codes for viruses, oncogenes) [20]. As previously mentioned, another major challenge was polymer size control during raw material manufacture. Firstly, the applied high temperatures tend to break the long deoxyribonucleotide polymer into smaller chains (i.e., even if the polymer is thermoresistant). For example, Placentex^®^ has an average MW of 350 kDa [21]. Secondly, these protocols cannot allow for precise control of the finally obtained MW. This aspect is potentially the reason why several reports describe PDRN as being a polymer with a MW range between 50 and 1500 kDa [2,4,9], which is quite a wide range for a raw material.

Since the first publications on PDRN, there has been an evolution of both PDRN sources and extraction protocols. Due to their relatively simple purification process, PDRNs and PNs are mainly collected from sperm cells of various species of salmon, mainly rainbow trout (*Oncorhynchus mykiss*) and chum salmon (*Oncorhynchus keta*) [2,9,49]. In fact, spermatozoa are the most appropriate cells to provide highly purified DNA with limited risks of impurities (e.g., peptides, proteins, and lipids), which can remain as contaminants from the processing of somatic cells [9]. Of note, it is documented that human mitochondrial DNA shares a 64.1% similarity with *Oncorhynchus mykiss* DNA [60], which brings some benefits. Specifically, Proskurina et al. (2023) [12] compared the biological properties of DNA extracted from human placenta, porcine placenta, and salmon sperm, concluding that the latter stimulated the maturation of dendritic cells, while having no effect on their allostimulatory capacity [12]. This means that PDRNs and PNs of salmon origin would have lower chances of eliciting an immune response, compared to polymers of human or porcine origin. In fact, numerous in vitro works and clinical studies have proven the biocompatibility and safety of DNA fragments of salmon origin [2,23,25,47,61,62].

However, PDRN/PN products tend to be relatively expensive, with the availability of the raw material depending on salmon breeding seasons [2]. Thus, alternative marine organisms have been successively studied as potential sources of DNA fragments, such as the starfish (*Patiria pectinifera*) [33] and sea cucumber sperm (*Apostichopus japonicus*) [63]. Furthermore, to overcome the ethical issues associated with animal-sourced raw materials, other natural sources such as red algae (*Porphyra* sp.) [47] or plants (*Panax ginseng*) [64] have been studied and were proven to have abundant reserves of PDRN [2]. The extraction and purification processes related to these different sources have been extensively reviewed by Nguyen et al. (2024) [2], as well as their advantages and disadvantages.

Notably, in comparison with salmon DNA, these alternative sources tend to have a slightly higher amount of protein content but are still effective in increasing cellular proliferation and in stimulating collagen production [33,47]. Furthermore, in vitro studies have reported their antioxidant efficacy and anti-inflammatory effects [47,63]. Similar actions to those of salmon PDRN were also shown for PDRN of plant origins, with effective regeneration results in in vitro skin cell models and in an artificial skin model [64].

Of further note, there is a tendency among different PDRN/PN suppliers for the trademarking of their extraction process (i.e., which is often also patented) and to name the corresponding raw materials accordingly. The most well-known examples (i.e., most frequently found in the literature) are described in Table 1.

Finally, there seems to be a correlation between the evolution of PDRN/PN extraction processes with the size of the obtained DNA fragments. Indeed, while PDRN was initially described as having a MW of 50 to 1500 kDa [2,4,9], a recent patent mentions a DNA fragment mixture corresponding to a MW of up to 10,000 kDa [11] (i.e., more than three times longer than previously reported MWs). Similarly to HA, where the size of the polymer determines its properties, the MW of the extracted DNA fragments might also confer different properties, which will be further discussed in this review (see Section 5).

## 5. PDRN/PN Molecular Weights Linked to Properties and Actions on the Skin

As previously mentioned, PDRN has been traditionally described as having a MW of 50 to 1500 kDa [2,4,9], which corresponds to a relatively wide range of polymer sizes. However, PN has been recently introduced by several reports, describing this polymer as composed of 13 covalently linked nucleotide monomers with a high MW of up to 8000 kDa and a viscoelastic texture [67,68]. Furthermore, a recent patent mentioned that the described preparation can comprise a mixture of PDRN and PN, where the DNA fragment mixture may be characterized by a MW range of 50 to 10,000 kDa, with the PDRN having a MW of 50 to 2000 kDa [11]. Thus, in the described application, one can assume that the MW of PN corresponds to a range between 2000 kDa and 10,000 kDa. Overall, it is clear that a standardized classification for PDRN/PN based on polymer sizes is currently lacking, even though PN is generally mentioned as a “high-molecular-weight” polymer [22,23,24].

In general, different polymer sizes lead to different physicochemical characteristics and potentially to different therapeutic actions. Even when focusing specifically on the aesthetic field, there are several examples of such MW-based function variability (e.g., HA and keratin). For example, a study comparing two keratin hydrolysates, with respective MWs of 3.58 kDa and 12.4 kDa, concluded that the hydrolysate with a lower MW had a higher impact on accelerating epidermal turnover, while the sample with a higher MW was more efficient at restoring dermal strength through the action of fibroblasts [69].

Similarly, it is well known that the MW of HA greatly influences its activity. Low-MW HA (i.e., 5–10 kDa) stimulates keratinocytes to produce CD44, which leads to epidermal proliferation and differentiation, while medium- and high-MW HA (i.e., 200–500 kDa and 1.5–2.0 MDa) are more efficient at improving skin hydration [70,71]. Moreover, it was shown that high-MW HA potentiates the differentiation of human monocytes into fibrocytes, while low- and medium-MW HA inhibit fibrocyte differentiation [72].

Thus, one can deduce that different MWs of DNA fragments can also influence their physiological actions in the skin. Of note, Hwang et al. (2018) classified various sizes of DNA fragments as follows: (i) low-MW PDRN (<50 kDa), (ii) medium-MW (“classic”) PDRN (50–1500 kDa), and (iii) high-MW PDRN (>1500 kDa) [31]. The study concluded that the apparent surface wound healing processes were not significantly different between PDRN molecular sizes [31]. However, the medium-MW (“classic”) PDRN group revealed less lipid accumulation with increased collagen composition and increased cell migration [31].

It is important to note that, as discussed in Section 3, the efficacy of the wound healing process promoted by DNA fragments directly depends on PDRN/PN polymer degradation. The deoxyribonucleotides (i.e., units that compose the polymer) are the chemical entities which actually stimulate the A_2A_ receptors in fibroblasts, thereby triggering the regeneration process. Thus, it is logical that (i.e., when directly injected in the wound site) all DNA fragments may potentially be able to stimulate skin regeneration, due to their natural local breakdown by endogenous nucleases. Several clinical studies have attested to the effectiveness of PDRN in skin regeneration, as reviewed by Colangelo et al. (2020) and Nguyen (2024), as well as the clinical benefits of PN, extensively reviewed by Lee et al. (2024) [2,6,73].

Besides being skin regeneration promoters, PDRNs/PNs are known as hydrophilic polymers [74], capable of absorbing water. From a biological viewpoint, the entire surface of a DNA molecule is covered by water, which interacts with the phosphate groups through hydrogen bonds [7,75,76]. Specifically, DNA hydration is fundamental for structural maintenance and functional integrity [77,78,79]. Therein, a study revealed that for several polynucleotides (e.g., DNA, RNA, etc.), there are two molecules of water per nucleotide (i.e., for the “monolayer content”) [80]. Thus, the water content of these polymers depends on their length. Based on these considerations, it may be set forth that in the aesthetic field, long DNA fragments (i.e., PN) will have higher hydration power than shorter polymers (i.e., PDRN), thus presenting superior attributes in terms of restoring the skin’s hydrobalance. In fact, a recent consensus paper by Italian practitioners clearly evidenced the benefits of PN in increasing skin hydration in their daily practice [25].

Furthermore, due to its high MW and hydrophilic characteristics, PN has been used to produce hydrogels, or PN fillers, with high viscosity values [24]. Such products can be injected into the skin to restore lost volume [1,25,45,81], while also stimulating skin cells due to the deoxyribonucleotides that are released during PN filler natural degradation by nucleases (see Section 3). Therein, a consequent benefit of PN over PDRN is its residence time in the skin, since PN corresponds to a long chain of nucleotides. Thus, its degradation by nucleases will require more time in situ, which means that PN would promote a longer skin stimulation effect, with potentially enhanced benefits for the patients.

Importantly, the polymer MW to be used in a final product should also be selected by taking the formulation parameters into account. As mentioned before, PN or medium-MW (“classic”) PDRN are preferred to make hydrogels or dermboosters for injection. Contrastingly, for topical formulations such as creams or gels, low-MW PDRN would be preferred. As an example, low-MW PDRN (i.e., extracted from seaweed), with a MW < 50 kDa, has been patented for skin and tissue permeation [82], as well as PDRN of 1 kDa or less to improve skin permeation for topical applications [10]. The latter appears to improve skin permeation rates compared to medium-MW (“classic”) PDRN [10]. Furthermore, a recent patent describes a skin-permeable composition containing a DNA fragment mixture for skin aging and regeneration, with the condensing of the DNA fragment mixture (i.e., 50 to 10,000 kDa) into monodisperse particles by adding a cationic additive (i.e., particles with charges of 0 mV to –30 mV). This processing element is important because long polymer chains cannot be absorbed into the skin [11]. In contrast, the obtained particles should achieve permeation, while maintaining long chains of nucleotides for a prolonged action.

**Figure 4:**
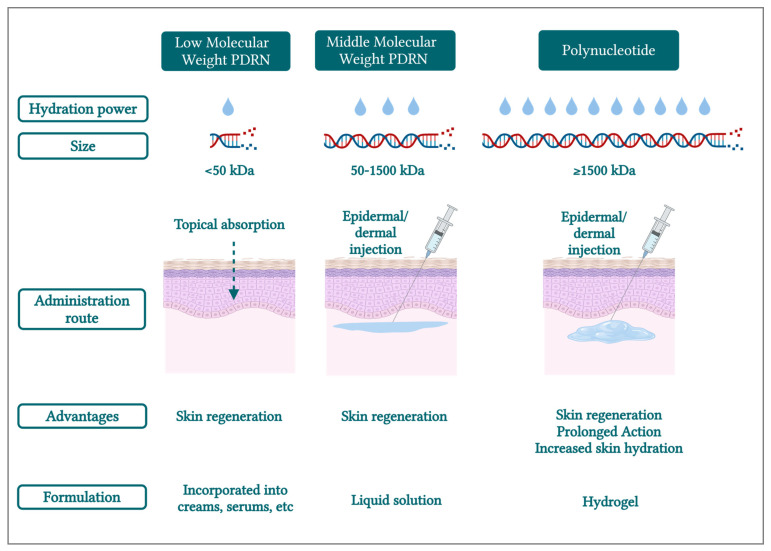
Molecular weight ranges, potential applications, and expected clinical benefits of PDRN- and PN-based formulations and products [31]. Based on polymer size, the optimal administration route may vary. kDa—kilodalton; PDRN—polydeoxyribonucleotide; PN—polynucleotide.

In conclusion, the MW cutoff of 1500 kDa which was set forth herein with the “Marques Polynucleotide Cutoff” is the most scientifically supported threshold for distinguishing PDRN and PN in aesthetic formulations. This cutoff is based on studies that classify PDRN within the 50–1500 kDa range [9,31] and PN as having higher MWs, often exceeding 2000 kDa. While clinical data directly comparing PDRN and PN are limited, consensus studies highlighted the efficacy of high-MW PN for addressing fine lines, directly reinforcing the relevance of this 1500 kDa threshold [25]. Overall, by establishing the “Marques Polynucleotide Cutoff” threshold value, this review notably aims to simplify future comparisons of literature results regarding PDRN and PN efficacy. Thus, based on the information discussed herein and on recent reports, PDRN and PN terminology should be used (i.e., for aesthetic product formulation purposes) according to their MW class, as described in Figure 4 and in accordance with the above-defined “Marques Polynucleotide Cutoff” of 1500 kDa.

## 6. Properties and Potential Applications of PN-Based Hydrogels

Modern extraction processes for PDRN have enabled us to obtain molecules of higher MW, maintaining their safe characteristics (i.e., low immunogenicity and residual level of proteins) [6,9,83]. Polynucleotides are polyanionic, hydrophilic, and polyelectrolytic natural biomaterials that can absorb large amounts of water, mostly via H-bond interactions [75]. Notably, the ability of PN to attract water enables it to form PN-based hydrogels. Such formulations offer many desirable attributes, making them an ideal choice as a transient implantable biomaterial for diverse human clinical applications. Indeed, DNA-based hydrogels show biodegradability, biocompatibility, modularity, non-toxicity, and hydrophilicity [75]. Consequently, while initial aesthetic formulations mainly included liquid PDRN solutions, several commercial PN-containing hydrogels are now available on the market. Therein, products usually contain higher concentrations (i.e., around 20 mg/mL) and higher chain lengths of PDRN (i.e., which is referred to as PN); however, the exact chain size is usually undisclosed [23,45,59,68].

A recent study by Kim et al. (2024) [22], comparing a DOT-PDRN solution and DOT-PN hydrogel microscopic structures (i.e., in SEM), revealed an amorphous structure for PDRN and a scaffolded structure for the PN-based hydrogel [22]. The latter was organized in regular polygons, which can achieve tessellation (i.e., triangles, squares, or hexagon) structures [22], with sizes ranging from 1 to 7 μm [22], confirming PN’s potential to form scaffolds. Another interesting study evaluated the 3D cellular invasion behavior of DNA hydrogels using 3D spheroid culture models. The authors concluded that the DNA scaffold supplemented with collagen was conducive to the highest cellular invasion and in situ cell proliferation, suggesting that the hybrid composition represents the best ECM composition and mimics a better ex vivo environment for cells [84]. The study concluded that DNA-based hydrogels, either individually or in combination with other biocomponents, have the potential for real-world applications, including tissue engineering, controlled drug release, cell therapy, and biosensing, to name a few [84].

In terms of aesthetic applications, PN-based hydrogel efficacy is primarily correlated with its hydrating action [23,45,59,68]. Furthermore, it is known that PDRN is degraded by unspecific plasma DNA nucleases, or by nucleases bound to cell membranes, leading to the formation of oligo- and mononucleotides [85]. Thereby, PDRN degradation gives rise to the formation of nucleosides and nucleotides that become available for exerting the main activity of the compound (i.e., by binding to the adenosine A_2A_ receptor and supplying the salvage pathway). Importantly, a liquid formulation of PDRN will be degraded faster than a PN-based hydrogel, since the surface area available to the nucleases in the latter is much smaller than that of a liquid PDRN formulation. Thus, a PN-based hydrogel will present slower degradation kinetics, while ensuring prolonged skin stimulation, for sustained results. Of note, several clinical studies conducted with PN-based hydrogels (i.e., 20 mg/mL, MW not mentioned) have shown an increase in skin hydration and elasticity parameters, without adverse effects, even in sensitive areas such as the periorbital area and labia majora [23,59,68,81]. These results may be linked to the fact that DNA-based hydrogels show biodegradability, biocompatibility, modularity, non-toxicity, hydrophilicity, self-healing attributes, and the ability to probe, program, and reprogram diverse biological systems [75].

Generally, hydrogels have been widely used in aesthetic medicine to mechanically support ECM reconstruction. They have a similar network topology and water content to the ECM, thus having emerged as the most popular implantable scaffolds [86,87]. Hydrogel formulations (i.e., mainly HA fillers) are probably among the most-used products in aesthetics. Besides being formulated with an endogenous molecule, HA fillers combine an immediate mechanical volumizing function with the hydration effects of HA [88,89,90,91]. Thus, some authors have been recently exploring the benefits of a combination of the regenerative action of PN with the physiological and mechanical actions of HA hydrogels.

A notable study compared two hydrogel formulations in terms of clinical efficacy on oral soft tissue healing, the first product containing PN (7.5 mg/mL) and the second containing PN + HA (10 mg/mL + 10 mg/mL) [32]. Unfortunately, the MWs are not mentioned in the report. The cell growth stimulation effects were similar between the two formulations, but cells in the PN + HA group tended to form nodules and stack up to form multilayers. The higher cell density achieved by the PN + HA formulation facilitated the formation of more numerous collagen-positive nodules with high cell density and multilayered cells, which corresponds to keynotes of ECM formation. Interestingly, the PN + HA formulation also ensured a faster gap closure, which was correlated with the hydrogel structure, since HA might improve the matrix structure, facilitating fibroblast migration [32]. Thus, the results suggested that in the case of the PN-based hydrogel formulation, its viscosity and structure might be more relevant than the PN concentration.

Globally, it has been shown that PN-based or PN-containing hydrogels act as structural scaffolds, bearing great promise in aesthetic medicine due to their hydrophilic nature and ability to mimic the ECM, promoting cell migration and endogenous ECM reconstruction. Furthermore, their effects are enhanced over time through natural in situ polymer degradation, stimulating A_2A_ receptors and the salvage pathway. Finally, combining PN with HA appears to further enhance the desired clinical outcomes.

## 7. Conclusions

PDRNs and PNs, as DNA-derived biopolymers, have shown significant potential in wound healing and skin regeneration applications. Due to their biocompatibility, PDRN- and PN-based formulations can be clinically used in sensitive areas, such as the periorbital area, which was previously mainly reserved for HA fillers. In parallel, these formulations can be clinically applied for the priming of other treatments, such as laser, radiofrequency, and LED protocols. Importantly, to better understand the clinical properties of this polymer class, there should be more transparency in published reports on the MW which is used, since it can greatly affect product properties. Moreover, this review proposed a novel structure-based nomenclature standardization for the terms “PDRN” and “PN” in aesthetic and cosmeceutical applications, with the defined “Marques Polynucleotide Cutoff” value of 1500 kDa. Thus, these terms should only be used as follows:

• “PDRN” (i.e., low-MW and medium-MW [“classic”]) for the description of small and medium chains of deoxyribonucleotides, with MW < 50 kDa and between 50 and 1500 kDa, respectively;
• “PN” should specifically refer to long chains of deoxyribonucleotides (≥1500 kDa).

It is also important to consider that even if manufacturers disclose the initial size of the DNA fragments (i.e., raw material as sourced from suppliers), PDRNs and PNs are not easily dissolved in water. This means that often, during the preparation of the aesthetic formulations, the polymer is subjected to high shear forces (e.g., vortexing, magnetic stirring, etc.) or to increased temperatures (i.e., warming the solution to accelerate the dissolution process, sterilization). However, if not properly controlled, the formulation process can degrade the DNA fragments even further, meaning that the final MW would be lower than the specified value. Thus, different formulations with the same concentration of PDRN/PN might have different viscosities, due to the effective final MW of the polymer, potentially resulting in different physiological actions in the skin. Finally, it is important to note that there is a significant economic difference in the commercial pricing of PN and PDRN. As with most biopolymers, longer chains are more challenging to produce due to the conservative nature of the manufacturing process. Consequently, injectable-grade PN consistently has a higher price (e.g., potentially up to twice as much) compared to injectable-grade PDRN.

## Figures and Tables

**Table 1:** Selection of trademarked processes and the associated PN/PDRN technologies mentioned in the literature. DNA—deoxyribonucleic acid; PDRN—polydeoxyribonucleotide; PN—polynucleotide.

Trademarked Process	Resulting Product	Registered Owner
DOT^TM^ (DNA Fragment Optimizing Technology) [65,66]	DOT ^TM^ PDRN/DOT ^TM^ PN	PharmaResearch Co., Ltd. (Gangneung, Republic of Korea)
HPT ^TM^ (Highly Purified Technology) [24,25]	Polynucleotide-HPT ^TM^, PN-HPT ^TM^	MASTELLI S.R.L. (Sanremo, Italy)

## Data Availability

Not applicable.

## Abbreviations

bp	base pairs
CaHA	calcium hydroxylapatite
DNA	deoxyribonucleic acid
ECM	extracellular matrix
IL	interleukin
kDa	kilodalton
LED	light-emitting diode
MMP	matrix metalloproteinase
MW	molecular weight
PCL	polycaprolactone
PDRN	polydeoxyribonucleotide
PLLA	poly-L-lactic acid
PN	polynucleotide
RNA	ribonucleic acid
ROS	reactive oxygen species
SEM	scanning electron microscopy
TNF	tumor necrosis factor
VEGF	vascular endothelial growth factor
